# A Novel Electromagnetic Response Measurement System for Continuous Monitoring of Meat Aging

**DOI:** 10.3390/foods14122016

**Published:** 2025-06-06

**Authors:** Dairoku Muramatsu, Yukino Sasaki

**Affiliations:** Department of Mechanical and Intelligent Systems Engineering, The University of Electro-Communications, 1-5-1 Chofugaoka, Chofu 182-8585, Tokyo, Japan; s2532044@edu.cc.uec.ac.jp

**Keywords:** meat aging, electromagnetic response, bioimpedance, non-destructive evaluation

## Abstract

The aging of dry meat enhances its flavor and tenderness; however, continuous internal quality monitoring throughout the aging process is challenging. We developed and validated a novel electromagnetic response measurement system for meat aging that enables continuous bioimpedance monitoring under stable, optimal temperature/humidity conditions. The system comprises a temperature-controlled dry aging fridge and a newly designed puncture-type semi-rigid coaxial probe, allowing for minimally invasive internal measurements over a broad frequency range. The probe achieved stable measurements across 10 kHz to 10 MHz, and its small diameter (1.25 mm) enabled almost non-destructive internal sensing. Beef and pork samples were monitored over 14 days via multi-channel bioimpedance measurements. After an initial stabilization period, bioimpedance steadily decreased throughout aging. This decline reflected progressive increases in tissue conductivity as cell membranes broke down and intracellular fluids leaked out. High-frequency measurements (e.g., around 10 MHz) were more sensitive to environmental disturbances. Periodic defrost cycles in the chamber caused temporary impedance dips at these frequencies, highlighting the influence of short-term temperature/humidity fluctuations. The system enables long-term continuous measurement without removing samples from the fridge, thus maintaining aging conditions during monitoring. Overall, the system enables the stable, long-term, and multi-channel electromagnetic monitoring of meat quality under optimal aging conditions—a capability not achieved in previous studies. This new method offers a minimally invasive, frequency-resolved approach for assessing meat quality evolution during aging. This advance demonstrates a new approach for tracking meat quality changes during dry aging.

## 1. Introduction

Packaged meat products at retail stores usually display information such as the origin, livestock species, cut, expiration date, and prices, which serve as important criteria for consumers. However, detailed attributes associated with the “deliciousness” (palatability) of meat—such as marbling, texture, flavor, and umami—are generally not provided. These attributes change significantly due to autolysis by the meat’s internal enzymes after rigor mortis (i.e., during aging); hence, it is important to evaluate the quality of meat during the aging process or in the aged state at the point of sale. Analyzing these qualities requires sensory evaluations by expert panelists with special discernment or physicochemical tests using large-scale equipment, which incurs substantial costs and time [1]. Therefore, a non-destructive and simple method for evaluating meat quality is needed [2,3].

Many attempts have been made to evaluate meat quality and freshness using engineering techniques. For example, non-destructive estimation of beef marbling scores using near-infrared hyperspectral imaging [4] and analysis of water mobility during the aging process using low-field NMR [5] have been reported. Additionally, evaluating the meat aging process—whereby muscle proteins are gradually broken down by endogenous enzymes (such as cathepsins and calpains) and surface microbes after slaughter, producing peptides and amino acids that improve umami, tenderness, and flavor—is also important. Regarding the physicochemical mechanisms of meat aging, knowledge is accumulating at the molecular level, for example through the correlation analysis of lipid oxidation and peptide formation contributing to flavor enhancement in dry-aged beef and analysis of the effects of aging temperature control on myofibrillar protein fragmentation patterns [6]. Furthermore, it has been revealed that the primary factor contributing to the improvement of meat texture during aging is the degradation of myofibrillar proteins and cytoskeletal structures by endogenous enzymes, particularly the calpain system [7]. Flavor enhancement is attributed to the release of free amino acids and peptides through protein breakdown, as well as byproducts from the degradation of adenosine triphosphate [8]. These compounds not only contribute directly to taste qualities such as sweetness, bitterness, and umami, but also serve as precursors to volatile aroma compounds through Maillard reactions and other processes during cooking, thereby influencing the aroma of meat [9].

In terms of studies based on the electrical properties of meat, a variety of approaches have been explored, including attempts to identify the breed and cut of beef using bioelectrical impedance [10], to predict the marbling score of beef using a portable impedance device [11], to estimate meat texture considering muscle fiber orientation [12], and to evaluate meat freshness using bioelectrical impedance analysis [13,14,15]. In recent years, research combining impedance measurements with machine learning has been reported for quantitative classification of the freshness of chilled meat, and a model using complex dielectric spectra to simultaneously estimate moisture and fat content has also been proposed, indicating growing interest in applying impedance measurements to multivariate analysis [16]. As for meat quality evaluation using machine learning, various approaches have been explored. These include comparative studies of algorithms such as k-nearest neighbors, support vector machines, and naive Bayes [17]; attempts to classify meat freshness by detecting gases released during spoilage using gas sensors and extracting their features for classification with support vector machines [18]; and methods using electronic nose systems to collect odor data from chicken meat, convert it into image format, and classify freshness with convolutional neural networks [19]. In addition, machine learning has been combined with other technologies such as imaging and spectroscopy, smart packaging, microbiological testing, and physical/chemical component analysis [20]. Furthermore, an IoT technique has been developed that integrates impedance measurement functionality into a radio frequency identification sensor tag to monitor the degree of aging in real time during distribution [21]. Detailed analyses of heating efficiency and temperature distribution in chicken meat using a 40.68 MHz radio frequency oven have also been conducted [22].

Thus, while attempts to evaluate meat quality through electrical characteristics have attracted significant attention, most previous studies have been limited to assessing specific parameters such as fat or water content. Another major issue is that few discussions have addressed the measurement environment (temperature, humidity) of the meat or the temporal changes that occur during measurement. In many prior studies, samples were taken out of the aging environment at set intervals for measurements in a laboratory, and fluctuations in temperature/humidity or probe repositioning for each measurement could greatly affect the results [23]. Moreover, the environmental conditions change with each measurement when measurements are performed outside of the aging fridge, making it difficult to strictly separate meat quality changes due to aging from the effects of environmental factors. In other words, no study has yet evaluated the electromagnetic response characteristics of meat over the long term in a stable manner while maintaining conditions suitable for aging (e.g., appropriate temperature, humidity, and hygiene). In this study, we developed a system capable of multi-faceted and detailed measurement of the electromagnetic response characteristics during meat aging and verified its basic performance using two types of meat samples (beef and pork).

## 2. Materials and Methods

### 2.1. Electromagnetic Response Measurement System for Meat Aging

**Figure 1:**
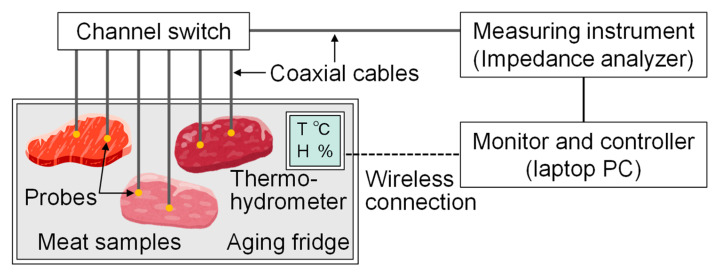
Electromagnetic response measurement system used during meat aging.

The electrical properties of meat are known to change with protein degradation and changes in water content as aging progresses [16,24], and it may be possible to evaluate meat quality by inputting electromagnetic waves using a probe that contacts or punctures the meat and measuring the response. Figure 1 shows the electromagnetic response measurement system used during meat aging proposed in this study. The system consists of an aging fridge, a thermo-hygrometer, probes, coaxial cables, a channel switch, and an electromagnetic response measuring instrument.

We adopted dry aging—primarily used for industrial meat aging—as the aging method, and installed a dedicated aging fridge (Landig + Lava GmbH & Co. KG, DRY AGER DX500P, Bad Saulgau, Germany). Dry aging is an aging method in which meat cuts are stored in an aerobic environment, at ~0–2 °C and 75–85% humidity, where muscle proteases (such as cathepsins and calpains) and surface microorganisms slowly break down myofibrillar proteins and connective tissue, increasing free amino acids and small peptides to enhance texture and flavor. Although surface drying and mold growth reduce yield, the recent availability of aging fridges with precise temperature, humidity, and airflow control has promoted hygiene and consistency, and demand for dry-aged meat as a high-value product is increasing.

The measurement probe attached to the meat inside the fridge is connected to the electromagnetic measurement instrument via coaxial cables and a channel switch. In this study, to evaluate responses over multiple frequencies, we used an impedance analyzer (Keysight E4990A, Santa Rosa, CA, USA) as the measuring instrument. We employed an 8-channel coaxial switch (Stack Electronics Co., Ltd., AV231, Akishima, Tokyo, Japan) on the probe side as the channel switch, which allows for easy electronic switching of the channel connected to the instrument. The internal circuitry of the switch does not simply toggle connections; rather, it maintains impedance matching for the selected channel to prevent unwanted signal reflections along the transmission path. The degree of high-frequency reflection due to impedance mismatch is defined by the voltage standing wave ratio (VSWR), and generally, a VSWR ≤ 3 indicates low reflection. We measured the input–output VSWR for each channel connection in the proposed system and found it to be 1.3 or below, confirming that the switch’s influence on high-frequency measurements is negligible.

**Figure 2:**
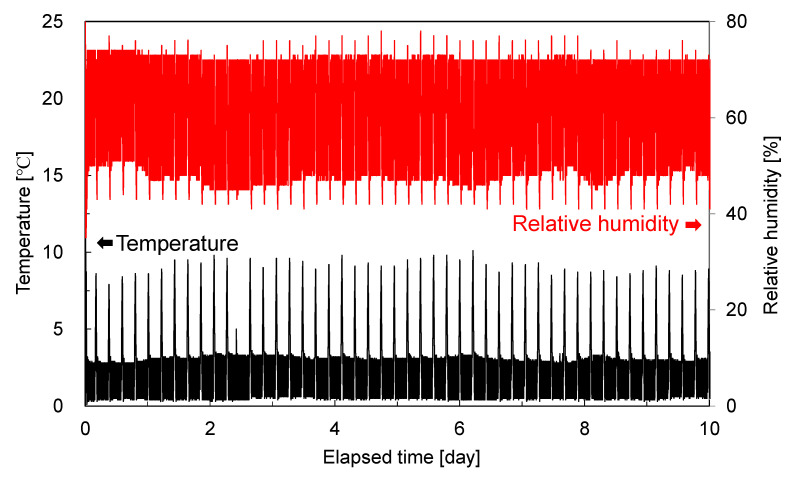
Temperature and relative humidity characteristics inside the aging fridge.

We measured the internal temperature and humidity characteristics of the fridge using a wireless thermo-hygrometer (T&D Corporation, TR-72wb, Matsumoto, Nagano, Japan) and found that they remained stable within the optimal range for aging, as shown in Figure 2. The temperature, recorded at 10 min intervals, averaged 2.6 °C (standard deviation of 0.8 °C), and the relative humidity averaged 64% (standard deviation of 4%), both remaining within the recommended range for dry aging (0–4 °C and 60–80%). The spatial variation in temperature and humidity inside the fridge was within ±0.2 °C and ±2% RH, confirming sufficient environmental uniformity. The internal lighting contained no ultraviolet components and does not influence the meat temperature. Additionally, a transient rise in temperature and drop in humidity observed approximately every 12 h were due to defrosting cycles; after each cycle, conditions quickly returned to the set points, confirming that electromagnetic measurements can be carried out under a stable environment with minimal disturbance.

### 2.2. Measurement Probes

**Figure 3:**
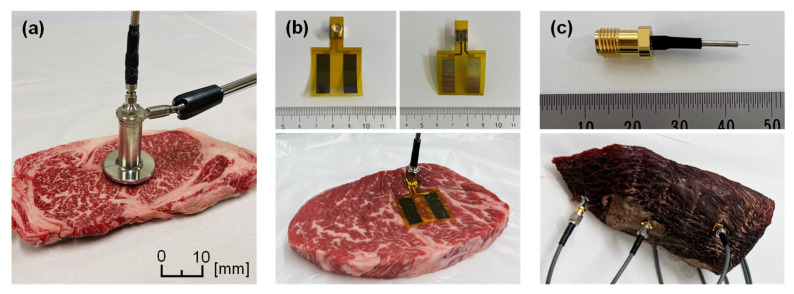
Probes for measuring electrical properties. (**a**) Open-ended coaxial probe; (**b**) surface attached-type flexible electrode probe; (**c**) puncture-type semi-rigid coaxial probe.

In the proposed meat aging system, a measurement probe that attaches directly to the meat is required. The authors previously investigated and developed electromagnetic phantoms that replicate the electrical properties of biological tissues such as muscle, fat, and skin, as well as probes for measuring their properties [25,26,27,28]. Figure 3a–c show representative probe types, and Table 1 summarizes their characteristics. The open-ended coaxial probe is mainly used for dielectric measurements at high frequencies, from a few hundred MHz to tens of GHz, and it is well-studied and easy to apply [29,30,31]. However, as it is designed for dielectric measurements, it is extremely expensive. Furthermore, it is difficult to measure the internal state of the meat due to the shallow sensing depth inherent to the open-ended coaxial method.

A surface-attached flexible electrode probe is suitable for use in the range of several kHz to tens of MHz [32]. It is easy to use, as it only needs to be attached to the sample surface and, using low frequencies, it can probe relatively deep into the meat. However, surface attached-type probes are sensitive to the interface conditions (contact area, moisture, etc.) between the probe and the sample [32,33]. The interface conditions can change significantly during long-term measurements; hence, this type of probe is not suitable for monitoring the aging process.

**Figure 4:**
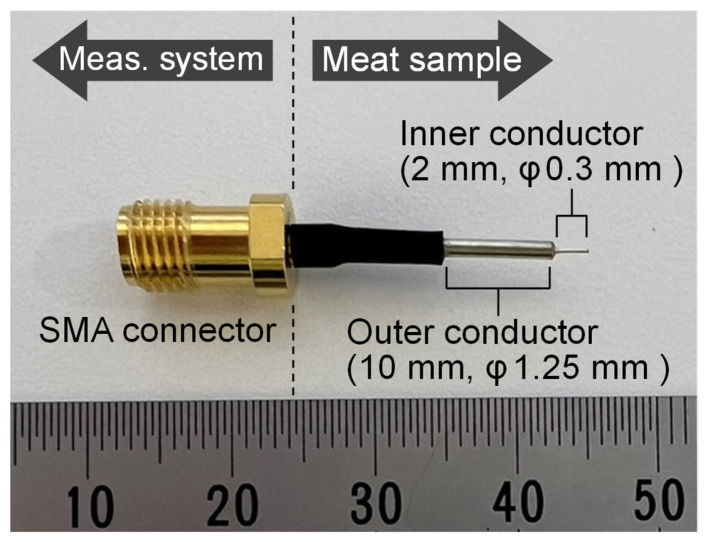
Puncture-type semi-rigid coaxial probe.

To enable stable internal measurements of meat by puncture in this study, we prototyped a puncture-type semi-rigid coaxial probe, as shown in Figure 4. Because this probe has a semi-rigid coaxial structure, it can be used stably over an extremely wide frequency range (from a few kHz to several GHz), and being puncture-type, it can measure the internal state of the meat and, thanks to a stable interface, is suitable for long-term measurement. Moreover, since it uses a general semi-rigid coaxial design, it can be manufactured inexpensively in large quantities. In the probe’s puncture section, the inner conductor protrudes by 2 mm and the outer conductor by 10 mm, leaving a 2 mm dielectric gap between them. The materials of the probe are as follows: the inner conductor is silver plated copper clad steel wire, the outer conductor is a tin plated copper tube, and the dielectric is solid polytetrafluoroethylene. The diameters of the puncture tip are approximately 0.3 mm for the inner conductor and 1.25 mm for the outer conductor, which are very small, enabling measurements that are almost non-destructive. When this probe is inserted into meat, a high-frequency electromagnetic field is generated between the inner and outer conductors, and the response varies according to the state of the meat.

### 2.3. Meat Samples

**Figure 5:**
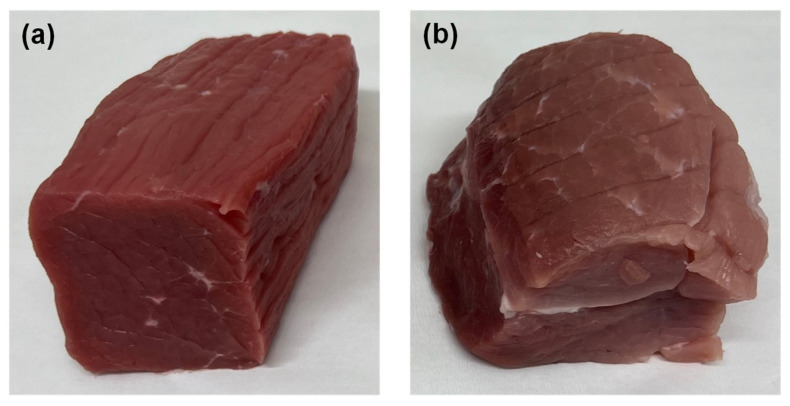
Meat samples. (**a**) Beef round; (**b**) pork leg.

Figure 5a,b show the meat samples used to evaluate the electromagnetic response measurement system; their details are given in Table 2. Two types of meat samples were prepared: (a) a block of beef round and (b) a block of pork leg. Both meat samples were purchased from local supermarkets and used in the experiments shortly after purchase. Sample (a) is predominantly lean with very little marbling, corresponding to a marbling grade of 1 (lowest) on the Japanese beef marbling standard (12-grade scale) [34]. Sample (b) is also mostly lean and similarly has almost no marbling. This study focused on the development and evaluation of a meat electromagnetic response monitoring system. To accelerate the aging process via higher water activity and enzymatic activity, lean meat with low marbling was selected as the sample. Each meat sample was trimmed to approximately 60 × 60 × 100 mm^3^ for the experiments. The weights of the trimmed samples were 314 g for (a) and 294 g for (b).

### 2.4. Measurement Parameters and Duration

Various parameters (such as reflection or transmission characteristics) can be used to measure the electromagnetic response of meat during aging; however, in this study, we adopted the absolute value of bioimpedance as the measurement parameter because it can be easily measured even with low-cost measurement ICs [35]. The electrical properties of biological tissues are known to depend on frequency [36,37], and the choice of measurement frequency is an extremely important factor in bioimpedance analysis [38,39,40]. A wide range of frequencies, from tens of hertz to the GHz band, has been used to evaluate food products’ electrical properties. For example, impedance spectroscopy over 50 Hz–1 MHz has been applied to yogurt quality inspection, and frequencies of 125 Hz–128 kHz have been used to evaluate meat freshness [41,42]. In addition, there are examples of utilizing high-frequency dielectric responses in the GHz range to assess the quality of chicken meat using microwave ring resonators or open-ended coaxial probes [43,44]. In this study, we focused on the intermediate frequency range [45] that is supported by readily available devices, and measured the bioimpedance of meat from 10 kHz to 10 MHz. The measurement signal level from the impedance analyzer was 500 mV, and 16 measurements were taken consecutively at each measurement point, with their average used as the representative value.

During dry aging, rapid breakdown of myofibrillar proteins by proteases causes tenderization to progress, and most major quality changes (including development of the characteristic dry-aged aroma) are essentially complete in approximately the first two weeks [46,47]. It has also been reported that the specific flavor compounds of dry-aged meat do not increase with aging beyond 21 days [48] and that there is little difference in flavor compounds between 3 weeks and 2 weeks of aging [49]. Beyond about 2 weeks, sensory improvements are nearly saturated, while only yield loss continues to increase significantly [50]. Based on these reports, the measurement period in this study was set to 14 days.

## 3. Results and Discussion

**Figure 6:**
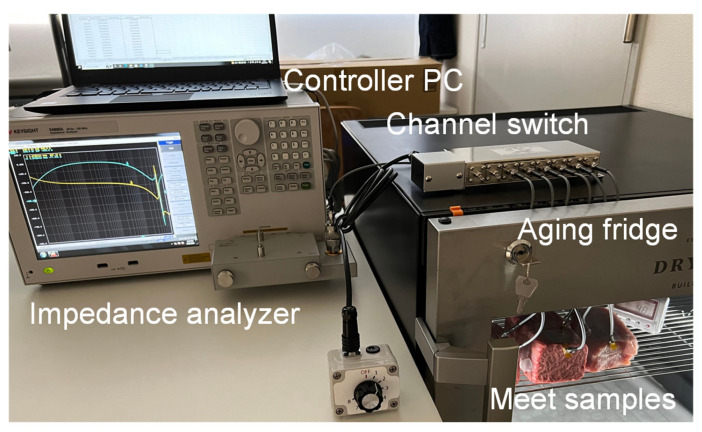
Setup for measuring electromagnetic responses of meat samples during the aging process.

**Figure 7:**
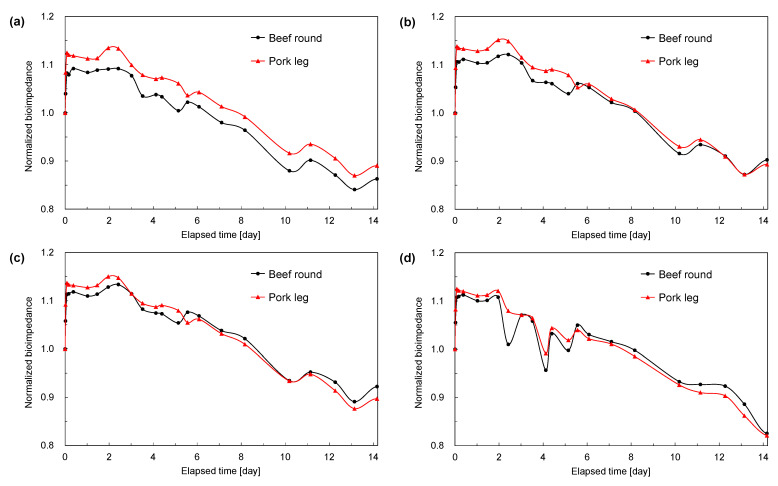
Bioimpedance of meat samples as a function of elapsed time (day). (**a**) 10 kHz; (**b**) 100 kHz; (**c**) 1 MHz; (**d**) 10 MHz.

To evaluate the proposed meat aging measurement system, we measured the bioimpedance changes in the two meat samples over 14 days at frequencies from 10 kHz to 10 MHz using the prototyped puncture-type semi-rigid coaxial probe. The measurement setup is shown in Figure 6. The bioimpedance results of each meat sample at 10 kHz, 100 kHz, 1 MHz, and 10 MHz are presented in Figure 7a–d. The vertical axis shows the bioimpedance normalized by the value of each sample at the start of measurement.

**Figure 8:**
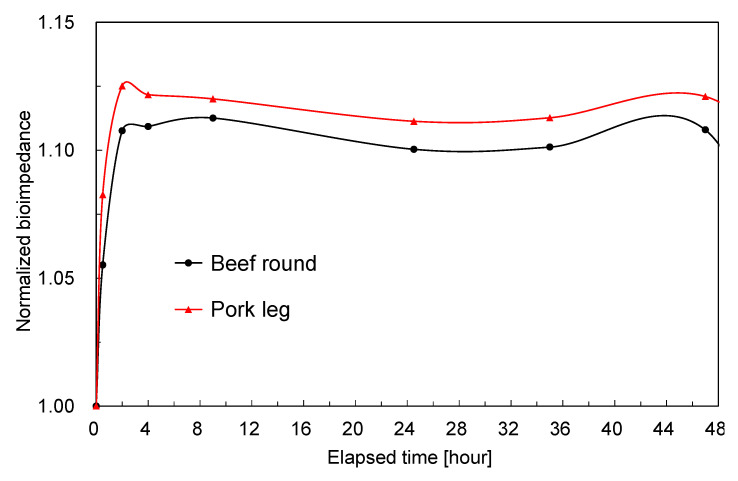
Bioimpedance of meat samples at 10 MHz during the first 48 h of measurement.

As shown in Figure 7, for all meat samples at all measured frequencies, the bioimpedance increased sharply just after the start of measurement. To illustrate this transient increase in more detail, Figure 8 shows the bioimpedance at 10 MHz, focusing on the first 48 h of measurement. Because this fluctuation occurs over only a few hours, it is not attributable to enzymatic autolysis within the meat (aging effect) or changes in water content; rather, it is thought to result from the instability of the interface between the probe and the meat immediately after insertion. Once a metal electrode contacts an electrolyte, bioimpedance measurements exhibit substantial drift in electrode potential and impedance during the first few tens of minutes due to active charge redistribution and double-layer formation at the electrode–tissue interface. These interfacial phenomena typically converge over tens of minutes to a few hours, after which electrode potential and impedance stabilize. The rapid bioimpedance changes observed at the start of our measurements can therefore be ascribed to these electrode potential fluctuations [51,52,53].

The overall trend observed over the 14-day aging period was that the bioimpedance decreased in both meat samples at all measurement frequencies. This is thought to be because, as aging progresses, cell membranes are destroyed and intracellular fluid is released, eliminating the equivalent capacitors formed by cell membranes, and current flows through the highly conductive intracellular fluid, reducing the bioimpedance along the path. The time-course of normalized bioimpedance at 10 kHz–10 MHz shown in Figure 7 exhibits a similar downward trend at all frequencies, indicating that in the intermediate frequency range, the processes of cell membrane collapse and conductive path reorganization during aging can be captured uniformly regardless of frequency. In addition, although the two samples were from different species (beef vs. pork), their bioimpedance change trends were the same. This is attributed to the fact that both samples had almost no marbling (i.e., were very lean), suggesting that the electrical properties of muscle and their temporal changes are not dependent on animal species.

**Figure 9:**
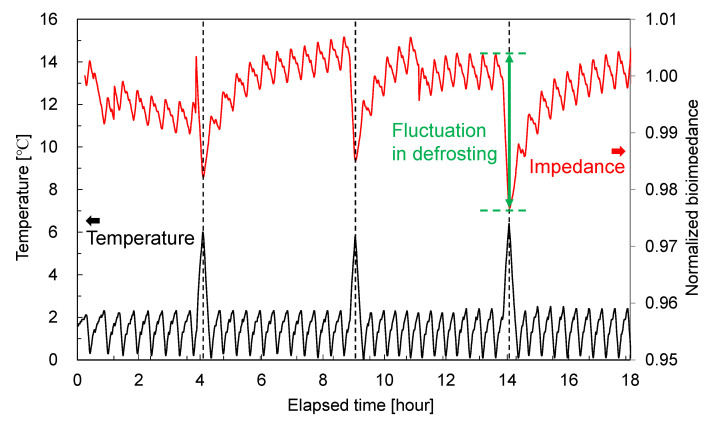
Bioimpedance of beef round during the defrosting of the fridge.

In the 10 MHz results shown in Figure 7d, distinctive drops in bioimpedance were observed on days 2, 4, and 5 that did not appear at the other frequencies. These changes deviate from the long-term aging trends and occurred at the same time for both meat samples; therefore, we considered that some disturbance in the aging fridge at those times caused short-term fluctuations in bioimpedance, and we conducted high-time-resolution measurements to investigate this effect. A probe was inserted into another round beef sample of a similar size (same dimensions as in Figure 5), and the bioimpedance at 10 MHz was measured at 1 m intervals for 18 h, along with monitoring the fridge temperature; the results are shown in Figure 9. We confirmed that the temperature changes were due to the fridge’s defrosting function. From Figure 9, it is evident that the timing of temperature increases in the fridge coincides with drops in bioimpedance; in other words, the short-term bioimpedance fluctuations were caused by changes in chamber temperature and humidity due to defrosting and the associated reversible changes in the meat’s state. This is presumed to be the cause of the characteristic bioimpedance dips observed in Figure 7d. Therefore, when taking measurements, it is advisable to use frequencies below 1 MHz, which are less affected by temperature changes, or to avoid taking measurements during defrost cycles. Furthermore, for practical aging monitoring, using a low-frequency band (below 1 MHz) and supplementing it with a high-frequency band, such as 10 MHz, to detect environmental fluctuations or apply temperature/humidity corrections could enable the evaluation of meat aging with reduced interference.

## 4. Conclusions

In this study, we developed an electromagnetic response measurement system—comprising a dry-aging fridge, coaxial probes, electrical measurement instruments, and related components—for the meat aging process. This system can maintain the recommended conditions for dry aging (0–4 °C, 60–80% humidity) while performing electromagnetic measurements using up to eight channels. To stably measure the internal quality of meat via puncture, we developed a novel puncture-type semi-rigid coaxial probe that can be used reliably over a wide frequency range. This probe enables the long-term stable measurement of the meat’s internal state and, with a puncture diameter of only 1.25 mm, achieves measurements that are almost non-destructive. To evaluate the developed system, we used it to measure the bioimpedance of two types of meat samples (beef round and pork leg) over 14 days at 10 kHz–10 MHz. Immediately after the start of measurement, a transient increase in bioimpedance occurred due to electrode polarization and ion reorientation; however, this subsided within about 2 h, after which the bioimpedance gradually decreased across all frequencies. This downward behavior of bioimpedance is thought to reflect the reorganization of conductive pathways as cell membranes collapse and intracellular fluids leak out during aging. Additionally, a short-term dip in bioimpedance was observed at 10 MHz, synchronized with the defrost cycles of the fridge, indicating that fluctuations in the fridge’s temperature and humidity have a pronounced effect at higher frequencies. These results show that frequencies below 1 MHz, which have low temperature dependence, are considered effective for evaluating the meat aging process. In summary, the usefulness of the developed meat aging electromagnetic measurement system was confirmed. The insights obtained through its evaluation provide valuable foundational information for achieving long-term, non-destructive meat quality management during aging, and are expected to contribute to future developments such as automated algorithms for determining the degree of aging. In future work, we plan to use the developed system with a larger number of meat samples to establish quantitative relationships between aging and electromagnetic response, accounting for individual variation and enabling statistical interpretation. As this study exclusively used very lean meat samples, future evaluations using high-marbling-grade beef and pork with thick fat layers remain an important task.

## Figures and Tables

**Table 1:** Comparison of three types of electromagnetic measurement probes.

Category	Open-Ended Coaxial Probe	Surface-Attached Flexible Electrode Probe	Puncture-Type Semi-Rigid Coaxial Probe
Destructiveness	Non-destructive	Non-destructive	Minimally destructive
Frequency range	Wide several MHz–several hundred GHz (probe-dependent)	Moderate several kHz–several tens of MHz	Wide several kHz–several GHz
Sensing depth	Shallow	Moderate	Deep
Main features	Well-studied and easy to apply, but expensive	Easy to apply, but strongly affected by contact conditions	Easy to apply, inexpensive, and less affected by contact conditions

**Table 2:** Characteristics of the meat samples used in this study.

Sample	Beef Round	Pork Leg
Origin	Australia	Japan
Marbling	Almost none	
Size	60 × 60 × 100 mm^3^ block	
Weight	314 g	294 g

## Data Availability

The original contributions presented in this study are included in the article. Further inquiries can be directed to the corresponding author.

## References

[1] Zhao X., Zhuang H., Yoon S.-C., Dong Y., Wang W., Zhao W. (2017). Electrical Impedance Spectroscopy for Quality Assessment of Meat and Fish: A Review on Basic Principles, Measurement Methods, and Recent Advances. J. Food Qual..

[2] Damez J.-L., Clerjon S. (2013). Quantifying and predicting meat and meat products quality attributes using electromagnetic waves: An overview. Meat Sci..

[3] Ashim B., Zhang Y., Kumar P., Smith J. (2020). Current Perspectives of Meat Quality Evaluation: Techniques, Technologies, & Challenges. Meat Qual. Anal..

[4] Kim J.-H., Kim D.-H., Ji D., Lee H.-J., Yoon D.-K., Lee C.-H. (2017). Effect of Aging Process and Time on Physicochemical and Sensory Evaluation of Raw Beef Top Round and Shank Muscles Using an Electronic Tongue. Korean J. Food Sci. Anim. Resour..

[5] Guo L.-Y., Shao J.-H., Liu D.-Y., Xu X.-L., Zhou G.-H. (2014). The Distribution of Water in Pork Meat during Wet-curing as Studied by Low-field NMR. Food Sci. Technol. Res..

[6] Setyabrata D., Cooper B.R., Sobreira T.J.P., Legako J.F., Martini S., Kim Y.H.B. (2021). Elucidating mechanisms involved in flavor generation of dry-aged beef loins using metabolomics approach. Food Res. Int..

[7] Dashdorj D., Amna T., Hwang I. (2015). Influence of Specific Taste-Active Components on Meat Flavor as Affected by Intrinsic and Extrinsic Factors: An Overview. Eur. Food Res. Technol..

[8] Joo S.-T., Lee E.-Y., Son Y.-M., Hossain M.J., Kim C.-J., Kim S.-H., Hwang Y.-H. (2023). Aging Mechanism for Improving the Tenderness and Taste Characteristics of Meat. J. Anim. Sci. Technol..

[9] Shi H., Shahidi F., Wang J., Huang Y., Zou Y., Xu W., Wang D. (2021). Techniques for Postmortem Tenderisation in Meat Processing: Effectiveness, Application and Possible Mechanisms. Food Prod. Process. Nutr..

[10] Altmann M., Pliquett U. (2006). Prediction of Intramuscular Fat by Impedance Spectroscopy. Meat Sci..

[11] Lepetit J., Salé P., Favier R., Dalle R. (2002). Electrical Impedance and Tenderisation in Bovine Meat. Meat Sci..

[12] Wang S., Zhang Z., Zhao X., Xiao X. (2024). Electrical Impedance Spectroscopy for Non-Destructive Meat Freshness Assessment. Discov. Food.

[13] Magwili G.V., Cruz F.R.G., De Pedro R.A.C., Evangelista R.L.C., Icaro K.P.G., Villarosa K.A. Non-Invasive Moisture Content Prediction and Characterization of Chicken Meat Freshness by Bioelectrical Impedance Spectroscopy. Proceedings of the 2019 IEEE 11th International Conference on Humanoid, Nanotechnology, Information Technology, Communication and Control, Environment, and Management (HNICEM).

[14] Nguyen H.B., Nguyen L.T. (2015). Rapid and Non-Invasive Evaluation of Pork Meat Quality during Storage via Impedance Measurement. Int. J. Food Sci. Technol..

[15] Leng Y., Zhang C., Gao Y., Wang X. (2024). Bio-Impedance Measurements for Meat Quality Determination of Pork Loins under Repeated Freeze-Thaw Treatments. J. Food Compos. Anal..

[16] Nouri H., Guermazi M., Kallel A.Y., Hao W., Kanoun O. Meat Freshness Assessment Based on Impedance Spectroscopy and Distribution of Relaxation Times (DRT). Proceedings of the 2022 International Workshop on Impedance Spectroscopy (IWIS).

[17] Arsalane A., Klilou A., Barbri N.E. (2024). Performance Evaluation of Machine Learning Algorithms for Meat Freshness Assessment. IJECE.

[18] Bhuiyan Z.W., Redwanul Haider S.A., Haque A., Hasan M., Uddin M.R. Meat Freshness Classifier with Machine and AI. Proceedings of the 2023 IEEE Region 10 Symposium (TENSYMP).

[19] Xiong Y., Li Y., Wang C., Shi H., Wang S., Yong C., Gong Y., Zhang W., Zou X. (2023). Non-Destructive Detection of Chicken Freshness Based on Electronic Nose Technology and Transfer Learning. Agriculture.

[20] Viancy V., Gobalakrishnan N., Anitha E. Advancements in Food and Meat Freshness Monitoring: Integrating Machine Learning and Advanced Technologies. Proceedings of the 2024 International Conference on Inventive Computation Technologies (ICICT).

[21] Muradov M., Cullen J., Mason A., Mukhopadhyay S.C. (2016). Real-Time Monitoring of Meat Drying Process Using Electromagnetic Wave Sensors. Next Generation Sensors and Systems.

[22] Goñi S.M., d’Amore M., Della Valle M., Olivera D.F., Salvadori V.O., Marra F. (2022). Effect of Load Spatial Configuration on the Heating of Chicken Meat Assisted by Radio Frequency at 40.68 MHz. Foods.

[23] Swatland H.J. (1989). Objective Measurement of Physical Aspects of Meat Quality. Proceedings of the Reciprocal Meat Conference.

[24] Damez J.-L., Clerjon S., Abouelkaram S., Lepetit J. (2008). Beef Meat Electrical Impedance Spectroscopy and Anisotropy Sensing for Non-Invasive Early Assessment of Meat Ageing. J. Food Eng..

[25] Muramatsu D., Koshiji F., Koshiji K., Sasaki K. (2014). Development and Study of Electrical Property on Phantom for Human Body Communication Considering Tissue Structure of Human Arm. Trans. Jpn. Inst. Electron. Packag..

[26] Muramatsu D., Koshiji F., Koshiji K., Sasaki K. (2015). Multilayered Phantom for Input Impedance Evaluation of Human Body Communication Electrodes. EAI Endorsed Trans. Cogn. Commun..

[27] Muramatsu D. (2021). NaCl-Based Blood Phantom Analysis for In Vitro Bioimpedance Measurement. AIP Adv..

[28] Muramatsu D., Sasaki K. (2022). Bioimpedance Emulation Performance of Multilayered Phantom for HF-Band. IEEJ Trans. Electron. Inf. Syst..

[29] Stuchly M.A., Stuchly S.S. (1980). Coaxial Line Reflection Methods for Measuring Dielectric Properties of Biological Substances at Radio and Microwave Frequencies-A Review. IEEE Trans. Instrum. Meas..

[30] Liu S., Fukuoka M., Sakai N. (2012). Dielectric Properties of Fish Flesh at Microwave Frequency. Food Sci. Technol. Res..

[31] Anand G., Lowe A., Al-Jumaily A. (2019). Tissue phantoms to mimic the dielectric properties of human forearm section for multi-frequency bioimpedance analysis at low frequencies. Mater. Sci. Eng. C.

[32] Muramatsu D., Sasaki K. (2021). Input Impedance Analysis of Wearable Antenna and Experimental Study with Real Human Subjects: Differences between Individual Users. Electronics.

[33] Muramatsu D. (2021). Bioimpedance-Based Plethysmogram Detection Using MHz Band. IEEJ Trans. Electr. Electron. Eng..

[34] Japan Meat Grading Association (2016). Beef Carcass Grading Standards (12-Grade Marbling Scale).

[35] Analog Devices (2012). AD5933: High Precision Impedance Converter Network Analyzer Data Sheet.

[36] Foster F.C., Schwan H.P. (1971). Dielectric properties of tissues in the 10 Hz to 20 GHz range. IEEE Trans. Microw. Theory Tech..

[37] Gabriel S., Lau R.W., Gabriel C. (1996). The dielectric properties of biological tissues: II. Measurements in the frequency range 10 Hz to 20 GHz. Phys. Med. Biol..

[38] Schwan H.P. Electrical properties of tissues and cell suspensions: Mechanisms and models. Proceedings of the 16th Annual International Conference of the IEEE Engineering in Medicine and Biology Society.

[39] Takamatsu R., Higuchi K., Muramatsu D. Measurement Frequency Evaluation for Bioimpedance-Based Blood-Glucose Estimation. Proceedings of the 2021 IEEE 3rd Global Conference on Life Sciences and Technologies (LifeTech).

[40] Robin A., Levkov K., González-Díaz C., López-Saquisilí N., Golberg A. (2024). Electrical bioimpedance spectroscopy as a non-invasive monitoring tool of physiological states of macroalgae tissues: Example on the impact of electroporation on 8 different seaweed species. Eur. Food Res. Technol..

[41] Kitamura Y., Toyoda K., Park B. (2000). Electric Impedance Spectroscopy for Yogurt Processing. Food Sci. Technol. Res..

[42] Huh S., Kim H.-J., Lee S., Cho J., Jang A., Bae J. (2021). Utilization of Electrical Impedance Spectroscopy and Image Classification for Non-Invasive Early Assessment of Meat Freshness. Sensors.

[43] Jilani M.T., Wen W.P., Cheong L.Y., Ur Rehman M.Z. (2016). A Microwave Ring-Resonator Sensor for Non-Invasive Assessment of Meat Aging. Sensors.

[44] Trabelsi S. (2015). Variation of the Dielectric Properties of Chicken Meat with Frequency and Temperature. Food Meas..

[45] European Commission Scientific Committees (2015). Intermediate-Frequency Electromagnetic Fields. *Champs Électromagnétiques* (Layman’s Guide). https://ec.europa.eu/health/scientific_committees/opinions_layman/fr/champs-electromagnetiques/l-2/6-intermediate-fields.htm.

[46] Dashdorj D., Tripathi V.K., Cho S., Kim Y., Hwang I. (2016). Dry Aging of Beef; Review. J. Anim. Sci. Technol..

[47] Ellies-Oury M.-P., Guéguen B., Deschamps M., Picard S., Sabatier P., Hocquette J.-F., Astruc E. (2022). Evolution of Sensory Properties of Beef during Long Dry Ageing. Foods.

[48] DeGreer S.L., Hunt M.C., Bratcher C.L., Crozier-Dodson B.A., Johnson D.E., Stika J.F. (2009). Effects of Dry Age of Bone-In and Boneless Strip Loins Using Two Aging Processes for Two Aging Times. Meat Sci..

[49] Smith R.D., Nicholson K.L., Nicholson J.D.W., Harris K.B., Miller R.K., Griffin D.B., Savell J.W. (2008). Dry versus Wet Aging of Beef: Retail Cutting Yields and Consumer Palatability Evaluations of Steaks from US Choice and US Select Short Loins. Meat Sci..

[50] Laster M.A., Smith R.D., Nicholson K.L., Nicholson J.D.W., Miller R.K., Griffin D.B., Harris K.B., Savell J.W. (2008). Dry versus Wet Aging of Beef: Retail Cutting Yields and Consumer Sensory Attribute Evaluations of Steaks from Ribeyes, Strip Loins, and Top Sirloins from Two Quality Grade Groups. Meat Sci..

[51] Schwan H.P. (1992). Linear and Nonlinear Electrode Polarization and Biological Materials. Ann. Biomed. Eng..

[52] Martinsen Ø.G., Grimnes S. (2014). Bioimpedance and Bioelectricity Basics.

[53] Barsoukov E., Macdonald J.R. (2018). Impedance Spectroscopy: Theory, Experiment, and Applications.

